# Vitamin D Deficiency and Its Role in Pathologies of Oxidative Stress: A Literature Review

**DOI:** 10.7759/cureus.90042

**Published:** 2025-08-13

**Authors:** Philippe Dentino, Johanna Mora, Li Zuo

**Affiliations:** 1 Department of Medicine, The University of Texas Rio Grande Valley School of Medicine, Edinburg, USA; 2 Department of Internal Medicine, The University of Texas Rio Grande Valley School of Medicine, Edinburg, USA; 3 Department of Physical Therapy, The University of Texas Rio Grande Valley School of Medicine, Edinburg, USA

**Keywords:** antioxidant, deficiency, oxidative stress, pathology, vitamin d

## Abstract

Vitamin D (VD) is a key mineral providing critical modulation of reduction-oxidation homeostasis within biological systems. VD and the VD receptor coordinate essential cellular activities, including mitochondrial electron transport, respiration, antioxidation, gene regulation, and calcium transportation. Reactive oxygen species (ROS), a natural by-product of cellular metabolism, play a prominent role in the mechanisms of biological antioxidant systems and form the basis of antioxidant therapies. However, ROS may also perpetuate pathological processes when produced in excess secondary to inflammatory pathways. Aging processes within cells rely on this reduction-oxidation homeostasis. Thus, the interaction with VD, its receptor, mitochondria, and oxidative stress forms the basis of nearly every known degenerative pathology, citing examples summarized throughout this review. Recent literature has highlighted the increasingly prominent role of VD deficiency as a unifying factor within the ubiquitous degenerative changes that ultimately progress these conditions discussed, such as metabolic syndrome, brain disease, cardiovascular disease, GI disease, immune dysfunction, musculoskeletal disease, and other age-related disorders. Lastly, the paradox of bacterial survival under lethal oxidative stress conditions is presented to explore the proposed mechanisms of such activity and the consideration of antioxidant therapies in the future.

## Introduction and background

Vitamin D deficiency (VDD) is a common and relatively understudied condition thought to affect up to an estimated 40% of the population of the United States and 26% of the population of Europe [1]. Until 1998, a deficiency of bodily vitamin D (VD) was defined as a blood serum level of 25-hydroxyvitamin D (25(OH)D) less than 10 ng/mL (25 nmol/L) [2,3]. However, global regulatory bodies have proposed definitions using < 50 nmol/L when considering population health, as the actual adequate physiologic value of VD continues to be studied [4]. Causes of VDD are broadly categorized by etiology of impaired synthesis, intake, metabolism, or absorption and linked to numerous metabolic, neurologic, immunologic, and musculoskeletal disease processes. Impaired synthesis of VD is mainly attributed to inadequate exposure to sunlight, often due to prolonged indoor residence or living above or below extreme geographic latitudes. Decreased nutritional intake of VD is another common etiology of VDD, as seen in individuals following restrictive dietary practices or in exclusively breast-fed infants. Impaired metabolism of VD is associated with physiologic dysfunction due to liver, renal, or immune disease, or interaction with medications relying on cytochrome P450 regulation. Bodily absorption of VD is crucial to supplementing nutritional VD intake and intrinsic VD synthesis. Poor VD absorption may be primarily due to malabsorptive GI conditions or secondary to the pathologies that impair the production or uptake of VD [5]. Although external manifestations of VDD typically do not present until extreme or persistently low levels of VD accrue, hypophosphatemia in the setting of osteomalacia is one notable mineral disturbance that may serve as an early marker of VDD and resistance to VD receptors (VDRs) and is seen in pathologies altering VD uptake, synthesis, or metabolism [6-8]. Other common conditions, including obesity [9-11], immunodeficiency [12-14], multiple sclerosis [15], irritable bowel syndrome [16,17], fibrocystic breast changes [18], cardiovascular disease (CVD) [19-21], menopause [22,23], chronic kidney disease [24], hepatic disease [25], sleep disorders [26], dental disease [9,27], and skeletal bone disease [28-30] have been associated with VDD. Similar physiological ties that underlie these disease processes can be linked to metabolic derangements in oxidative stress, described further in this text. Given the impactful and broad physiologic effects of VDD upon bodily systems, it is crucial to understand the role of VD in mechanisms of cellular oxidative stress and the pathological conditions associated with its deficiency. Moreover, conceptualizing the connections between VDD and pathologies of oxidative stress is essential when treating mineral deficiencies and the global burden of chronic degenerative diseases.

VDD and oxidative stress

VDD has been increasingly linked to oxidative stress and the progression of degenerative pathologies. Oxidative stress, characterized by an imbalance between reactive oxygen species (ROS) production during typical cellular respiration and antioxidant defense mechanisms, plays a pivotal role in cellular aging processes. ROS are primarily produced in mitochondria as byproducts of oxidative phosphorylation and act as intermediates between enzymes of many cellular defense mechanisms, including superoxide dismutase (SOD), lipid peroxidase, and glucose-6-phosphate dehydrogenase [31]. As VD is known to also interact with these cellular antioxidative enzymes, the VDR plays an equally critical part in these physiological pathways. The VDR is a transcription factor uniquely modulated by the active form of VD,1,25(OH)₂D₃, and manages calcium homeostasis, immunity, and cellular growth regulation by influencing the expression of a diverse set of genes [32]. While its primary role involves coordinating calcium metabolism for bone health, the VDR also directs immune cell differentiation, proliferation, and function. Additionally, VDR exhibits anticancer properties by leveraging intrinsic mechanisms of immunity to detect and eliminate aberrant cells, highlighting its integral involvement in maintaining physiological balance across bodily tissues and organ systems [33].

Oxidative stress and oxidative phosphorylation

Mitochondria are major endogenous sources of ROS due to their integral role in cellular oxidative phosphorylation [34]. Cellular metabolism occurs primarily within the mitochondrial membrane and is mediated through the electron transport chain (ETC) [35]. The ETC is an essential biochemical pathway occurring within the inner mitochondrial membrane, responsible for oxidative phosphorylation. This process generates adenosine triphosphate (ATP), and thus cellular energy, by transferring electrons through a series of protein complexes, ultimately reducing oxygen (O₂) to water (H₂O). The ETC not only provides an electromotive force to support metabolic functions but also creates a proton (H+) gradient to drive ATP synthesis. The process involves five key complexes, which function synergistically to harness the electromotive force [36]. ROS are a separate, yet also crucial, adjunctive electron source created during this process of mitochondrial oxidative phosphorylation [35]. Degradation of hydrogen peroxide (H₂O₂) through the ETC process becomes a driver of cytosolic messaging and subsequent cascades in addition to loss of electrons from ETC complexes I and III under hypoxic conditions [37,38], release of pro-apoptotic cytochrome-c from mitochondria [39], and forkhead box O transcription factor promotion of cell cycle arrest [40]. Although vital for cell function and survival, overactivation of these pathways leads to mitochondrial membrane leakage and dysfunction, which promotes excessive cellular oxidative stress and damaging macro-effects of ROS aggregation. Over time, the ubiquitous function of the ETC and its products becomes a prominent source of cellular aging and degenerative pathologies seen within every biological organ system.

Age-related decline in oxidative stress

**Figure 1:**
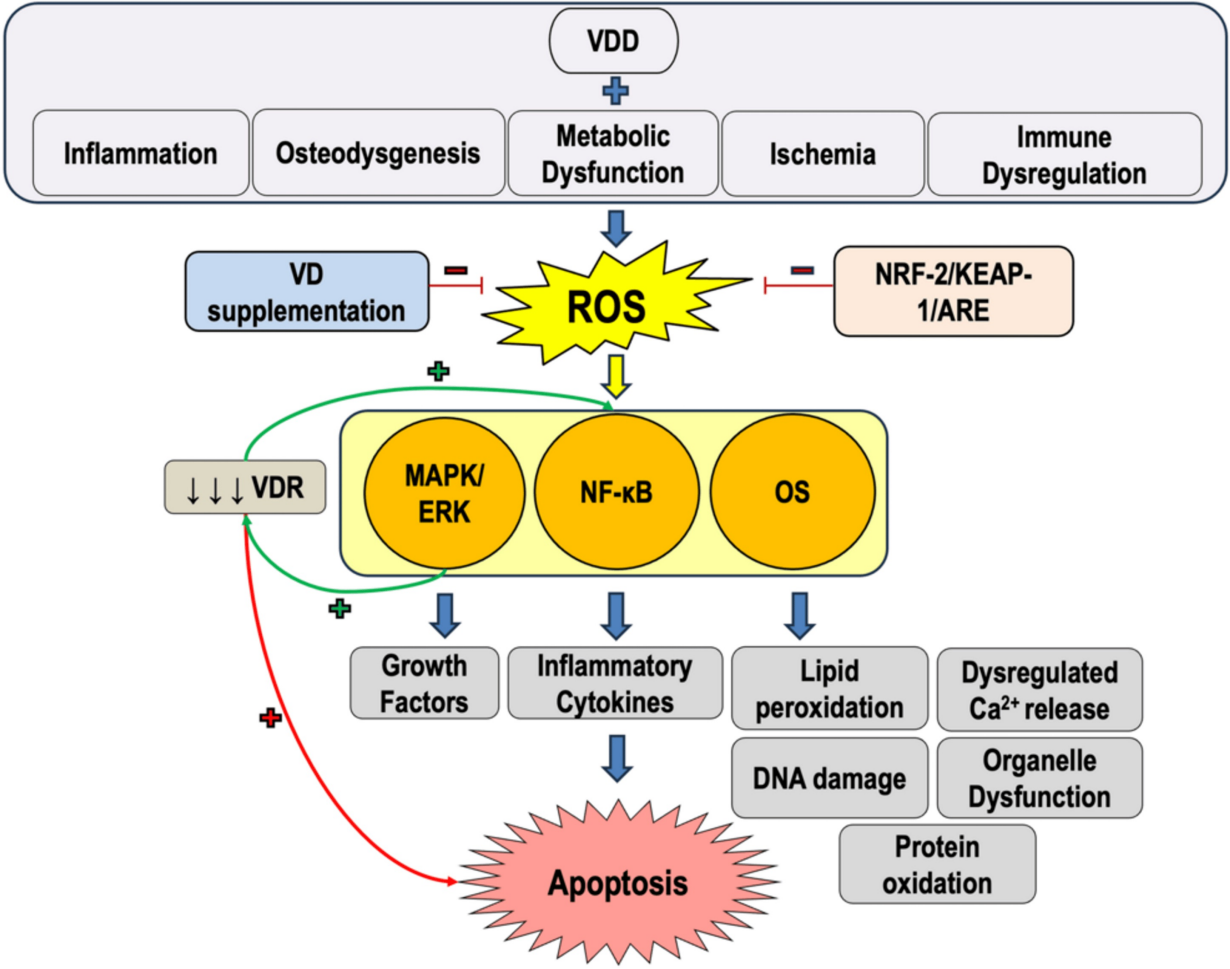
Diagram illustrating the relationships between VDD, inflammatory cellular changes, and ROS ARE, antioxidant response element; ERK, extracellular signal-regulated kinase; KEAP-1, Kelch-like ECH-associated protein 1; NF-κB, nuclear factor kappa B; NRF-2, nuclear factor (erythroid-derived 2)-like 2; OS, oxidative stress; VD, vitamin D; VDD, vitamin D deficiency; VDR, vitamin D receptor Image credit: Philippe Dentino

Aging is a complex, inevitable biological process that incurs degenerative cellular changes and is strongly influenced by genetic, environmental, and lifestyle factors [36]. Oxidative stress and overproduction of ROS have been identified as significant contributors to the aging process, both on a cellular level and an organ system level [34]. Accumulation of ROS within intracellular structures (such as the mitochondria and the endoplasmic reticulum) leads to oxidative damage and disruption of metabolic signaling pathways, including nuclear factor kappa B (NF-κB), mitogen-activated protein kinase (MAPK), nuclear factor (erythroid-derived 2)-like 2 (NRF-2)/Kelch-like ECH-associated protein 1 (KEAP-1)/antioxidant response element (ARE), and PI3K/Akt [34,41]. Disruption of these pathways may trigger apoptosis, delay replication, initiate hyperproliferation, or induce autophagy, thus leading to the irreversible oxidation of key cellular structures (Figure 1). Aggregate oxidative insult to these cellular structures over time may also lead to proteinaceous abnormalities, maladaptive cellular senescence, and organelle dysfunction. Although the scope of the enzymatic involvement in these occurrences is not completely understood, in this review, several enzymes are thoroughly examined within the context of cellular senescence and degeneration, discussed by their significance in the progression of pathologies of oxidative stress.

NF-κB

NF-κB is a nuclear transcription factor that regulates key physiologic inflammatory and immune responses. It has been found to be activated in response to ROS, products of oxidative stress, inflammatory signal cascades, and other biochemical stressors, leading to gene transcription that further these processes during proliferative and apoptotic cellular activities (Figure 1). Found in all biological organ systems, the prolonged activation of NF-κB has marked effects on the cell cycle and is implicated in both pro-apoptotic pathways [42] and antioxidant enzymatic activation [43]. NF-κB activation in myocytes has been associated with muscle atrophy (via the ubiquitin-proteasome system), impaired myocyte repair and regeneration, and upregulation of proinflammatory cytokines: tumor necrosis factor-alpha (TNF-α), IL-6, lipoxygenase (LOX), vascular cell adhesion molecule 1 (VCAM-1), and inducible nitric oxide synthase [36]. LOX may play a particular role in the generation of ROS in skeletal muscle tissue through its indirect interaction with prostaglandin synthesis and release of arachidonic acid (AA), which in turn is regulated by phospholipase A2 (PLA2) [44]. Although the exact pathway by which ROS interacts with AA and PLA2 is incompletely understood, heat-induced physiologic stress within murine skeletal muscle tissue has been shown to increase levels of extracellular ROS secondary to increased AA metabolic activity [45].

Extracellular calcium concentration regulation, which acts closely with VD and the VDR, has also been implicated in these pathways when ROS are generated within myocytes [45]. The direct relationship between VDD and NF-κB is less understood; however, NF-κB-mediated inflammation due to VDD demonstrated subsequent apoptosis and autophagy of intestinal epithelial cells (Figure 1) [46]. Conversely, alimentary VD supplementation demonstrated improved markers of glycemic control in insulin-resistant rodents [47] and platelet aggregation in patients living with type 2 diabetes mellitus (T2DM) [48]. These findings may suggest a potential role of NF-κB manipulation for therapeutic purposes, such as cancer therapies and immune therapies utilizing VD as an intermediate. Increases in NF-κB cytokine levels have been observed in pro-inflammatory, high oxidative stress conditions after selective reduction of VDR expression in tuberculoid macrophages, as well as in in situ experiments studying the activation of T and B lymphocytes [49,50]. These experimental trials provide several recent examples describing the complex interplay between inflammatory cytokine pathways, ROS, and VD, with the potential to be further explored for precise genetic manipulation therapies. Together, these studies illustrate the multifaceted role of NF-κB and its relationship with VD-mediated oxidative stress.

MAPK/ERK

MAPK is a family of phosphorylating proteins that initiate cellular responses to oxidative stress and inflammation, particularly in skeletal muscle. Importantly, MAPK serves as an actuator of growth factors within endocrine functions (IGFR), vascular proliferation (VEGF), hematologic differentiation (PDGFR), collagenous stimulation (EGFR), and cellular replication through extracellular signal-regulated kinase (ERK) and tumor suppressant p38. Additionally, MAPK regulation plays a central role in autophagy and apoptosis through interaction with Jun amino-terminal kinase (JNK) and p38 signaling, with subsequent phosphorylation of glycogen synthase kinase 3 beta (GSK3β) or caspases 3, 8, and 9. Within the context of the musculoskeletal system, MAPK activation balances myocyte adaptation (e.g., growth) and maladaptation (e.g., atrophy) depending on the context and intensity of the physiologic stimuli [36]. MAPK involvement in the generation of ROS has been explicitly linked to serine-threonine protein kinase activity through apoptosis signal-regulating kinase 1 (ASK1) and the JNK/p38 signaling mechanisms. ASK1 and interactions with TNF-α during cellular apoptosis are regulated by superoxide and peroxide ROS through their inhibition of JNK pathways, which cause downstream blockade of the dissociation of thioredoxin from the ASK1 complex [45]. Similarly, ROS-mediated tissue injury was modeled by the infliction of segmental amputations in *Schmidtea mediterranea* thoraxes, in which a reduction in MAPK/ERK complex activity was observed with decreased hydrogen peroxide concentration when a ROS signaling inhibitor was introduced [51]. Notably, these observations have been experimentally recreated in other non-human models, supporting the role of ROS in ERK-mediated tissue growth [52]. Important to note within this context is the cytotoxic role of the VDR, ROS, and the mitochondrial membrane in which these processes primarily occur (Figure 1). A study examining silencing of VDR expression in cells and measurement of extracellular levels of cytochrome c led to the conclusion that increased mitochondrial membrane permeability was linked to higher ROS concentrations and apoptotic death of the cells within 14 days of VDR removal [53]. Transcriptional control of the VDR has also been shown to influence cytotoxicity, which itself has been related to ROS buildup and modulation in cancer cells [54]. Establishing the proliferative function of the MAPK pathways and their influence on the VDR upon cellular apoptosis has brought forth additional studies investigating potential treatment options and refinement of current therapies to develop more closely controlled ROS-mediated antitumor cell therapeutic agents and materials.

NRF-2/KEAP-1/ARE

NRF-2 is a major regulator of the endogenous cellular antioxidant response. This nuclear transcription factor promotes ROS suppression through binding with inhibitor protein KEAP-1 and subsequent activation of ARE-dependent genes. In skeletal muscle, genetic upregulation of ARE leads to production of antioxidant enzymes, such as glutathione peroxidase 1 (GP-1), SOD, and catalases, which all neutralize ROS and provide cellular defense against oxidative disruption (Figure 1). In addition to these vital antioxidant systems, macro-interventions such as calorie intake restriction, intermittent fasting, selective dietary habits, and aerobic exercise have demonstrated beneficial effects in counteracting cellular aging processes. Biotherapies like senotherapy (targeted pro-aging cell senescence inhibition), sirtuin-activating compounds, and naturo-pharmacotherapies have been proposed to enhance cellular antioxidant activities, as well as promote biogenesis for the regeneration of necessary organelles to maintain physiologic homeostasis [55]. VD and the VDR are known to provide additional antioxidant properties synergistic with NRF-2 signaling pathways. These effects have been observed through Silent-mating-type information-regulation-2 homolog-1 (SIRT1) activation of NRF-2 antioxidant cascades in the presence of VD administration in murine cells when chronically exposed to galactose [56]. Downregulation of VDR expression and secondary reduction in NRF-2 antioxidant activity have also been demonstrated in patients with chronic obstructive pulmonary disease [57] and linked to other inflammatory conditions that involve NRF-2 pathways in neural, renal, and hepatic tissues [58,59]. As administration of VD has been linked to beneficial effects in the management of these diseases and other severe inflammatory conditions, NRF-2 remains a similarly valuable therapeutic target and area of study when considering the antioxidative relationship between VD and NRF-2.

The role of VD in modulating pathologies of oxidative stress

VDD has been associated with impairments of every biological organ system, many of which are implicated in degenerative, chronic, and pro-inflammatory conditions. These conditions are most frequently observed in the neurological, cardiovascular, GI, genitourinary, renal, and musculoskeletal systems, yet the pathology of impaired metabolism, immune response, and sleep is also impacted. Although some of these disease processes and associated pathophysiology are not fully understood, many demonstrate notable relationships to VDD and have been successfully targeted with the addition of therapeutic VD in experimental and clinical settings (Figure 1). The following sections will detail these pathologies seen within these organ-based and physiologic systems alongside their relationship with oxidative stress and VDD.

## Review

Neurodegenerative and psychiatric diseases

VDD has been linked to neurodegenerative conditions, such as Alzheimer’s disease (AD), Parkinson’s disease (PD), Huntington’s disease (HD), amyotrophic lateral sclerosis (ALS), and spinocerebellar ataxia disease. These conditions present with a wide range of cognitive impairments, neuromotor dysfunctions, and physiologic disturbances [60]. AD and PD are both progressive neurodegenerative disorders characterized by the loss of dopaminergic neurons, deposition of abnormal proteins within neuronal structures, and dysfunctional neurotransmitter production and regulation [61]. The pathophysiology is believed to occur through intracellular ROS accumulation, resulting in neuroinflammation and chronic accumulation of intracellular ⍺α-synuclein in PD and extracellular fibrillar β-amyloid protein (Aβ) in AD. Over-accumulation of these neurofibrillary plaque-forming proteins may ultimately incur neuronal death [62]. The role of ROS in the development of these neurodegenerative diseases has been more strongly associated with neuronal apoptotic processes, including Aβ-enhancement of calcineurin activation, leading to B-cell lymphoma 2 promoter release of mitochondrial cytochrome c and caspase activation [63]. As mitochondrial function is crucial to neuronal survival, dysfunction of lysosomal degradation of damaged mitochondria, inhibition of SOD1, and loss of substantia nigral polyunsaturated free fatty acids due to ROS exposure have also been distinctly implicated in the progression of HD, ALS, and PD, respectively [64].

Guanine oxidation by ROS had also been theorized to play a role in the CpG methylation activity of neuronal DNA, which supports the pathogenesis of plaque formation in AD [65]. VD and the VDR are thought to play a role in the modulation of aggregation of abnormal neuronal proteins, thus implicating the VDR as a target of neuronal disease management and further study [55]. Noting the implications of chronic metabolic and vascular disease on the neurological system, recent studies demonstrated that inadequate blood serum levels of VD in elderly adults with AD and PD were associated with more significant cognitive decline and earlier onset of dementia [66]. At the same time, individuals with T2DM had a higher prevalence of cognitive impairment if deficient in VD [67]. A rodent study examining VD-deficient murine neurons demonstrated increased production of Aβ and phosphorylation of Tau protein when antioxidant enzyme expressions of SOD1, GP-4, and cysteine-glutamate exchanger were inhibited, further supporting an antioxidant role of VD in neurons [68].

VDD also reduces antioxidant capacity in mice with mitochondrial dysfunction and induced expression of GSK3β, which has been associated with impaired synaptogenesis [61]. Conversely, VD supplementation has been associated with neuroprotective effects. Behavioral and neuromotor symptoms of PD induced through 6-hydroxydopamine in mice were blunted after administration of VD. Brain-derived neurotrophic factor, an inhibitor of neuronal apoptosis and dopaminergic degradation, was similarly observed to increase with the administration of VD [61]. Regarding the effects of ROS on neuronal survival and implications upon the pathogenesis of many of these neurodegenerative conditions, we speculate that the impact of VD may be beneficial in buttressing aberrant plaque protein accumulation in cerebral tissues and mitigating mitochondrial dysfunction in neurons.

Emotional lability, depression, suicide, and psychosis can develop after glucocorticoid treatment of multiple chronic inflammatory diseases, such as arthritis, asthma, or autoimmune disease [69]. These psychiatric symptoms are believed to occur secondary to excess exogenous glucocorticoid effects on the central nervous system. The mechanism behind these effects includes oxidative stress-induced nitration and poly (ADP)-ribosylation of cerebral proteins in the hippocampus [69]. Chronic administration of glucocorticoids also decreases VD levels due to modulation of VDR inhibition. VD supplementation in multiple experiments was shown to have reversible glucocorticoid neurotoxicity and antidepressive effects caused by chronic treatment [69]. The results of these studies support VD supplementation as an adjunctive therapy for patients requiring long-term glucocorticoid treatment to prevent oxidative stress associated with psychiatric conditions [69]. Attention-deficit/hyperactivity disorder (ADHD), a common behavioral disorder affecting 5-7% of school-age children worldwide, is a secondary example with similar underlying ROS pathology [70]. The etiology has not been fully identified; however, it is postulated that individuals living with ADHD have decreased neuronal levels of zinc and increased oxidative stress-mediated damage due to reduced activity of GP-1, hence, limiting cellular response to ROS buildup [71]. ADHD has also been associated with VD and magnesium deficiency, wherein supplementation of VD and magnesium for eight weeks improved behavioral function in children diagnosed with ADHD [70]. Autism spectrum disorder is another neurodevelopmental disease that has been linked to VDD during pregnancy [72]. Some evidence has been proposed showing that environmental factors and perinatal infections can insinuate maternal immune activation during pregnancy and subsequent placental vulnerability, leading to inflammatory and oxidative stress that is transferred to the developing brain of the fetus [73]. It is plausible then that the treatment potential of VD and antioxidant substrates presents a vital opportunity to alleviate the effects of oxidative stress contributing to these neurodevelopmental and psychiatric conditions.

CVDs

VDD has been implicated in numerous degenerative cardiovascular and endothelial conditions. Oxidative damage to vascular endothelial cells secondary to VDD is strongly associated with atherosclerosis [74], HTN, myocardial infarction (MI), and heart failure [75]. Furthermore, VD has been shown to modulate multiple critical antioxidant metabolic pathways in cardiac tissue [76,77]. Progression of atherosclerosis, HTN, and subsequent left ventricular hypertrophy has been observed in VDD individuals in the setting of chronic activation of the renin-angiotensin-aldosterone system (RAAS), increased expression of endothelial adhesion molecules (such as VCAM-1 and ICAM-1), foam cell formation, and vascular smooth muscle cell proliferation [78,79]. Increased vascular tone and impaired vasodilation from downregulation of endothelial nitric oxide synthase have also been measured in individuals with higher skin pigmentation, as VDD is thought to arise from inhibition of UV-mediated VD synthesis, hence exacerbating endothelial dysfunction [80,81]. In patients with ST-elevation MI, VDD has been associated with impaired epicardial reperfusion and increased thrombus burden following primary percutaneous coronary intervention [82].

Recent animal studies of MI models have provided evidence of the role of VD supplementation in mitigating myocyte fibrosis through the regulation of the TGF-β/Smad pathway [75,78]. Although observational studies have linked low VD levels with increased CVD risks, randomized controlled trials examining high-dose VD supplementation (>2000 IU/day) have shown mixed efficacy in the definitive reduction of major cardiovascular events [78]. Reperfusion of necrotic cardiomyocytes due to ischemic or traumatic insults has been thought to increase intracellular oxidative stress, triggering apoptotic mitochondrial permeability transition pore (mPTP) cascades (Figure 1) [83]. Optic atrophy 1 (OPA1)-mediated mitochondrial fusion within arterial smooth musculature has also been proposed as a mechanism through which endovascular damage is lessened in HTN via mitochondrial membrane cristae stabilization and sequestration of cytochrome c [84]. In the same murine model, OPA1 upregulation was associated with decreased ROS concentrations in the presence of mitochondrial fusion. Interestingly, experimental restriction of VD in mice has been associated with decreased skeletal myocyte respiration, although mitochondrial protein indicators of apoptosis or reduced respiratory function (i.e., OPA1) remained relatively unchanged [85]. Given the physiologic and anatomic complexity of the cardiovascular system, we surmise ROS and VD may be more intimately related in the pathogenesis of these CVDs when considering the effects of VDD, endothelial response, and the role of VD supplementation in mitigating cardiomyocyte oxidative stress.

Metabolic diseases

Metabolic health and function are closely tied with VDD, which collectively contributes to the pathogenesis and complications of chronic metabolic diseases such as T2DM and obesity [86]. VDD disrupts glucose homeostasis through inflammatory β-cell apoptosis, thereby impairing insulin secretion and contributing to the etiology of systemic insulin resistance [87]. In contrast, VD directly regulates pancreatic β-cell function through VDR-dependent calcium signaling, which is essential for insulin exocytosis [88], calcium flux through insulin-sensitive tissues, optimal glucose uptake, and reduced lipotoxicity [89]. In children, VDD has been linked with impaired thiol-disulfide homeostasis, a critical physiologic reduction-oxidation mechanism, which was shown to exacerbate oxidative damage seen in other inflammatory and disrupted metabolic states [90]. Administration of VD has been shown to reduce the secretion of TNF-α and IL-6, alleviating metabolic complications that arise with systemic insulin resistance [90,91].

Therapeutic targets of congenital metabolic conditions have been experimentally observed in individuals living with cystic fibrosis (CF), in which polymorphisms of the Bsml gene were shown to affect VDR affinity for its primary substrates, leading to increased markers of oxidative stress and inflammation [92]. Supplementation of VD in these individuals was later shown to reduce markers of inflammation (e.g., CRP and malondialdehyde (MDA)), supporting the usage of VD in treating patients for CF complications [93]. ROS accumulation and exposure are known to underlie many of these metabolic disease processes, owing to their integral involvement in protease regulation, neutrophil response, lipid metabolism, and production of oxidative intermediates, all associated with metabolic syndromes [94]. Chronic RAAS activation and insulin resistance, two hallmarks of long-standing metabolic dysfunction, are thought to occur through endothelial cell ROS release and hyperglycemia-induced NF-κB inflammation, respectively [95]. Palmitic acid-associated lipotoxicity has been studied in obesity, wherein ROS were hypothesized to worsen metabolic derangement when lipocytes could not store oxidative metabolites within saturated fatty acids [96]. In the context of conditions of metabolic dysfunction and the significant contributing effects of ROS, VD has shown promise in improving glycemic control and reducing markers of oxidative stress in individuals living with metabolic syndromes via reductions in HbA1c, fasting glucose, visceral adiposity, and markers of oxidative load.

Diseases of the immune system and genetic disorders

Maternal VDD has lasting effects on in utero fetal development, including reductions in CD4+ and CD8+ T cells and transcriptional downregulation in hematopoietic stem cells. These changes in innate immunity persist into adulthood, suggesting that inadequate prenatal VD exposure inhibits long-term “immunologic memory,” which increases the risk of immune-related conditions such as asthma and T2DM [97]. Obesity is another common metabolic and inflammatory disorder prevalent in modern children and adolescents [86]. In children with obstructive sleep apnea (OSA), VDD has been shown to worsen the comorbid effects of childhood obesity through adipose sequestration of VD, reducing its bioavailability [11]. Polymorphisms in the VDR gene have also been shown to significantly influence individual responses to VD supplementation, the effects of which may be seen in early childhood [93]. These observations have been tested with VD supplementation, after which pediatric patients with CF and BsmI polymorphisms exhibited clinically significant improvements in inflammatory and oxidative marker profiles with 4,000-10,000 IU/day of VD [92]. Adequate VD intake in childhood is well-established in literature for supporting healthy osteogenesis (Figure 1), bone density, and dental health [98]; however, in light of the profound implications of early exposure to adequate VD and detrimental outcomes of VDD in children, continued research is warranted to support clinical and theoretical guidelines for pediatric population health.

Considering the imbalance of oxidative stress in VDD, innate and acquired immune deficiencies are also influenced by the production and accumulation of ROS (Figure 1). States of high oxidative stress have been linked to increased activity of transcription factors such as NF-κB and hypoxia-inducible factor-1-alpha (HIF-1α), which induce cytokines and widespread inflammatory processes that strongly influence cellular immunity responses [99]. The study of ataxia-telangiectasia, a rare inherited disorder of impaired DNA repair caused by mutation of the ATM gene, has been studied as an example of innate immunity failure. These effects have been illustrated through the relationship between ROS accumulation and antioxidant protein systems (SOD and GSH), mitochondrial detoxification systems (NRF-1 and NADPH), and the NRF-2-KEAP pathway [100], leading to the immunocompromised state seen in individuals living with this condition [101]. Acquired immune deficiencies have also been connected with ROS generation. Non-steroidal anti-inflammatory drugs, which are frequently prescribed for common inflammatory conditions and injuries, have been used as a notable example of an exogenous ROS source that upregulates ROS-sensitive proteins and transcription factors, such as PPARγ, NF-κB, and AP-1, through inhibition of COX [99]. Antiretroviral therapies for HIV infection, a known disease progenitor of states of severe chronic oxidative stress, are another therapeutic agent that has been demonstrated to worsen oxidative homeostasis through incompletely understood mechanisms, but contribute to the overall mortality and morbidity of the HIV infection [102].

Sleep disorders

VDD has been studied in the context of disorders of sleep, fatigue, and metabolic conditions impairing homeostatic rest. Inadequate VD intake has been associated with both central and peripheral fatigue, characterized by dopamine and serotonin imbalance, which are critical for energy regulation and mood stabilization [103]. VD is understood to increase levels of nerve-growth factor, glial cell-derived neurotrophic factor, and p75NTR expression [104]. VD regulation of voltage-gated calcium and chloride channels is also known to contribute to motor neuron excitability and function, which may play a role in peripheral fatigue [104]. OSA, a sleep-related disorder characterized by repetitive, chronic episodes of oxygen deprivation (hypoxia) during sleep cycles, has been associated with low serum VD levels regardless of severity grade, although the pathophysiology is not completely understood [105]. In these patients, VDD was associated with poor cardiometabolic outcomes, elevated triglycerides, and high-sensitivity CRP [104]. Imbalances in oxidative stress and ROS production have also been attributed to clinical OSA presentation and pathogenesis severity. In individuals diagnosed with OSA, lower levels of HIF-1α and NF-κB, which reflect physiologic oxidative load under conditions of hypoxia, were experimentally correlated with at least two months of continuous positive airway pressure usage. Conversely, higher levels of VEGF, indicating endothelial proliferation and dysfunction, were associated with prolonged hypoxic conditions [106]. Moreover, cyclic hypoxia-reoxygenation is related to elevated oxidative stress markers, such as NADPH, ILs (IL-6 and IL-8), and lipid peroxidation [107,108].

Rodent models experimentally afflicted with manifestations of intermittent hypoxia (HTN, vascular remodeling, and MI) were observed to have marked reversion of these pathologic changes when exposed to antioxidant substances; furthermore, the exposed group failed to significantly undo the cardiometabolic changes in the associated tissues [109]. Other antioxidant therapies, including N-acetylcysteine, vitamin C, and leptin, have shown promise in reversing the adverse effects of OSA [106]. Bearing in mind the link between VD, ROS, and hypoxic sleep conditions, further investigation of targeted antioxidant control applied in these clinical scenarios can benefit individuals experiencing the deleterious outcomes of disordered sleep and OSA.

GI and hepatic diseases

VD and hepatic function are closely interrelated in physiologic function, as each contributes to systemic bodily detoxification and clearance of ROS. Metabolic dysfunction-associated steatotic liver disease (MASLD) is one of several complex pathological gastrohepatic conditions involving insulin resistance, lipid dysregulation, and chronic inflammation. VDD has been implicated as a risk in the development of MASLD; however, animal and human models have suggested variable upregulation and downregulation of VDR activity, rather than VD concentration, are responsible for the hepatocyte degradation and immune dysfunction observed as MASLD progresses [110]. Additionally, activation of the VDR in hepatocytes attenuates hepatic steatosis by reducing lipogenesis, promoting fatty oxidation, and inhibiting hepatic stellate cell activation, thereby preventing hepatocyte fibrosis [110]. In the GI system (gut-liver axis), VD modulates colonic microbiota and intestinal permeability, mitigating endotoxemia-driven hepatocyte injury by regulating tight junction proteins (e.g., claudins and occludins) and obstructing endotoxin translocation [111]. As seen with other fibrotic and degenerative hepatic diseases, VDD is prevalent in patients with hepatic cirrhosis, where deficiency levels correlate with worsened disease outcomes, higher Child-Pugh and MELD scores, and severity of hepatic encephalopathy [112]. Clinically, VD supplementation has shown potential in reducing ammonia-induced neurotoxicity seen in hepatic encephalopathy and enhancing outcomes in patients living with complications of chronic hepatic cirrhosis [113]. Inflammatory pathologies of the GI system share standard features with other systemic degenerative disease processes discussed previously in the review that involve oxidative stress imbalances, fibrosis, inflammation, and immune dysregulation. Inflammatory bowel disease (IBD), such as Crohn’s disease and ulcerative colitis, is a well-known example of chronic inflammatory intestinal disruptions due to mucosal immune dysfunction and oxidative stress.

Although the etiology of IBD is multifactorial, ROS accumulation exacerbates the oxygen gradient of the colonic intimal mucosa, thus disturbing the cellular metabolic homeostasis and creating the downstream effects of immune cell activation and proliferation within the luminal tissues [114]. Interestingly, HIF-1α and NRF-2 play a protective role in these conditions against ROS-mediated mucosal apoptosis; however, these effects are subjugated to lymphocytic infiltration and amplification of inflammatory cascades (IL-6, IL-8, and TNF-α), worsening the autoimmune destruction of the mucosal intima. In addition to the myriad of antioxidant defense mechanisms found within the intestinal mucosa, upregulation and overexpression of myeloperoxidases and matrix metalloproteinases (MMPs) compound the generation of ROS metabolites within mucosal cells and may interact with the natural gut microbiota and nutritional intake [115,116]. Potentially, VD intake or adjunct administration can provide benefits in reducing the oxidative imbalance within the GI mucosa and decreasing autoimmune activity in the intimal tissue.

Obstetric and gynecologic disorders

VD is vital to ovarian physiology and hormone regulation, particularly regarding the pathophysiology of preeclampsia and polycystic ovarian syndrome (PCOS). In patients experiencing hypertensive episodes in pregnancy, VDD has been shown to exacerbate oxidative stress, inflammatory cytokine production, and pro-vasodilatory responses, perpetuating the cyclic cascade of vascular dysfunction seen in severe preeclampsia [117]. This mechanism occurs via modulation of thromboxane/prostacyclin ratios, reduction of vasoconstriction, and slowing of systemic vascular endothelial damage [118]. VDD has also been associated with impaired folliculogenesis and elevated levels of advanced glycation end products, which in turn impair reproductive function [119]. Supplementation of VD in these cases can improve ovulatory function, reduce anti-Müllerian hormone levels, and modulate androgen production in individuals living with PCOS, contributing to the reduction of disruptive symptoms [120]. VD also influences granulosa cell function via microRNA regulation, which is linked to enhanced glucose uptake and reduced follicular cell apoptosis [121].

In breast, ovarian, and colorectal cancers, VD regulates tumor-promotor gene expression to reduce aberrant cellular proliferation and improve responses to cancer therapies, which may prevent cancer metastasis through regulation of epithelial-to-mesenchymal transition and ROS pathways [122]. A significant association between the ROS suppressant KEAP-1 and ovarian cancer was shown in vitro via genotyping single-nucleotide polymorphisms (SNPs) within KEAP-1 expressed from cancerous epithelial ovarian cells [123]. Although it is unknown whether SNPs directly alter the protein functionality of KEAP-1 to contribute to ROS imbalances, other associations between ROS and ovarian cancer likely exist, as evidenced by alterations in chemotherapeutic drug resistance, DNA damage, and tumor angiogenesis in these cells exposed to SNPs. Cellular ROS homeostasis additionally plays an important role in the expression of P-glycoprotein and multidrug resistance protein 1 in ovarian cancer cells. Programmed cell death ligand 1 and miRNA expression have been linked to DNA repair regulation and modulation of angiogenesis in ovarian tumors [124]. Chronic inflammatory states have also been connected to premature oocyte aging and subsequent fertility decline in the presence of ROS-mediated recruitment of IL-6 with CD4+ T cells, B cells, and microtubule degradation [125].

Musculoskeletal disease

VDD has been extensively studied in populations experiencing degenerative musculoskeletal conditions and has been closely linked to cartilage matrix breakdown secondary to increased states of oxidative stress [126]. In patients with knee osteoarthritis (OA), VDD was associated with biomarkers of oxidative stress in myocytes, such as increased MDA, increased total oxidant status, and decreased total antioxidant capacity. VDD was shown to accelerate the activity of MMP-1 and MMP-13, leading to the degenerative changes in cartilaginous tissue that characterize OA [127]. In experimental models of osteoporosis, osteoblast exposure to VD was shown to increase bone mineral density and improve cortical bone quality [128]. Furthermore, VD supplementation in ovariectomized rodents increased collagen production, decreased bone porosity, and improved lacunar microarchitecture within lumbar intervertebral discs and vertebral regions [128]. Longevity-associated gene SIRT1 is controlled partially by the effects of VD on the VDR, which has been associated with promoting chondrocyte survival and reducing cellular senescence in articular cartilage [129].

In models of induced VDD, reduced SIRT1 expression exacerbated cartilage degradation and bone loss, while VD supplementation restored SIRT1 activity, enhancing chondrocyte mitochondrial function and reducing markers of senescence such as p16 and p21 [130]. Disruption of ROS equilibrium and amplification of oxidative stress in collagenous tissue drive many of the underlying pathophysiologic processes causing bone demineralization, cartilage degeneration, and sarcopenia (muscular atrophy). Apoptosis of osteoclastic and osteoblastic cells and subsequent loss of bone mineral density in osteoporosis have been recreated under conditions of high oxidative stress, which is thought to occur from abnormal mitochondrial function in these cells, which require high levels of ATP to function [131]. Cartilaginous degradation related to oxidative stress shares many characteristics with other cellular processes of aging, albeit associated explicitly with the modified activity of MMPs, SOD, GSH, and VEGF in damaged or mechanically overstressed tendons, hence inducing inflammatory tissue changes that lead to the hallmark findings of tendon disruption (i.e., fibrosis, rupture, and calcification) [132]. Sarcopenia, a natural musculoskeletal aging process characterized by myocyte loss and shrinkage, has not been directly associated with oxidative stress; however, perturbation of the mitochondrial membranes and the mtPTP likely incurs organelle leakage of apoptotic cascade signal molecules, leading to the marked myocyte loss seen in sarcopenia [133].

Endocrine and integumentary diseases

VD plays an integral role in thyroid gland physiology. Thyroid hormone deficiency and downregulation of thyroid receptors have been identified as risk factors for developing thyroid disorders, although the current mechanisms theorized involve VD suppressing TSH-stimulated adenylyl cyclase activity, iodide uptake, and expression of DIO2, a key enzyme for converting T3 into T4 [134]. Although several studies have shown that increased VD concentrations are associated with lower TSH levels and Hashimoto’s thyroiditis, VD deficiency may also increase the risk of autoimmune thyroid disease due to the sensitivity of the hypothalamic-pituitary-thyroid axis. VD supplementation has also been significantly correlated to decreased rates of hypothyroidism [134]. Adding to the delicate thyroid hormone balance, the concentration of ROS within thyroid medullary and cortical cells is known as a crucial intermediate for gland-specific hormone synthesis [135]. Other thyroidopathies (disorders of the thyroid tissue or gland) have also been linked to altered mechanisms of oxidative damage in the gland. Hyperthyroidism is thought to contribute to inefficient antioxidant defense mechanisms within the thyroid and surrounding glandular tissue, producing oxidative stress [135]. In one rodent study, VDR-knockout murine models did not display reduced TSH levels as expected, highlighting that the mechanism of antioxidant VD effects within the thyroid may occur through unknown signaling pathways [134]. Low serum VD concentration has also been correlated with primary hypercortisolism (Cushing’s disease), an endocrine disorder characterized by abnormally high levels of serum cortisol, causing multiple systemic effects [136]. Similarly, individuals living with exogenous hormone-producing adrenal tumors displayed reduced serum levels of VD and decreased expression of the VDR [136]. In individuals living with hyperaldosteronism, VDD was also correlated with symptoms and systemic findings of increased RAAS activation, predominantly resulting in HTN [136].

ROS aggregation can have varied effects on the integumentary system at the organ, tissue, cellular, and molecular levels. ROS exposure to dermal cells has been shown to alter the structure of collagenous proteins, activate pro-inflammatory transcription factors, and upregulate the expression of EGFR in the skin, leading to inflammatory cytokine release and subsequent skin damage [137]. VD, which plays an additional role in stabilizing mast cells and attenuating the production of pro-inflammatory signals, has been associated with inhibition of the JAK-STAT pathway, production of IL-2, IL-7, IL-15, and IFN-gamma, and downregulation of activating ligands MICA and CXCL-10 in inflammatory pathways [138]. Alopecia areata, a condition characterized by patches of non-scarring hair loss on the scalp and thought to occur due to chronic activation of CD8+ T lymphocytes, has been linked to low serum VD [138]. Currently, topical VD derivatives are used to treat mild-to-moderate AA, but oral treatments have not been as strongly supported in clinical applications to aid in the treatment of severe AA [138]. Psoriasis, another systemic and immune-mediated condition manifesting as raised patches on the skin, has been successfully treated with VD administration, which has supported its use as a safe and effective adjuvant treatment for mild to moderate psoriasis. Though the exact mechanism is unknown, the therapeutic effects are believed to be related to NF-κB-mediated VD antioxidant activity [139]. In consideration of the organ systems discussed and the developing study of incompletely understood therapeutic pathways, VD supplementation appears promising in the clinical management and treatment of these inflammatory disorders.

The potential role of ROS in bacterial survival and VD exposure

The unique role of oxidative stress and bacterial survival remains an active area of study. The phenomenon of bacterial survival contingent upon intracellular ROS concentration was presented by Luan et al., examining the paradoxical growth of *Escherichia coli* populations at high quinolone concentrations (>1000 μmg/mL) [140]. Quinolones, a class of antibiotics that produce ROS through inhibition of DNA double-strand unwinding (via DNA gyrase) and subsequent DNA fracture, have been observed to cause an initial drop in* E. coli* survival as their concentration increases. Paradoxically, survival rates were shown to recover to nearly 100% at higher concentrations of quinolone products. ROS accumulation has been observed to peak at lethal quinolone concentrations (>400 μmg/mL) and decrease at high, paradoxically non-lethal levels (>1200 μmg/mL) [140].

This mechanism is believed to occur when high quinolone concentrations inhibit protein synthesis and suppress cellular respiration, leading to diminished ROS accumulation and reduced pro-apoptotic protein activity. Of note, moderate concentrations of quinolones allow sufficient ROS accumulation for cellular lethality, correlating with catalytic cycles in the ETC and quinolone-gyrase-DNA interactions. In vitro, nalidixic acid-mediated cellular death has been shown to be strongly ROS-dependent. As corresponding protein synthesis is required for adequate intracellular ROS accumulation, chloramphenicol was shown to block both protein synthesis and ROS accumulation, thus preventing cell death at moderate quinolone concentrations [140]. Later findings suggested mechanisms of oxidative stress may be externally manipulated, which challenged previous assumptions that direct DNA breaks from pharmaceutical products targeting DNA unwinding complexes are sufficient for bacterial death.

Hong et al. presented three theoretical mechanisms to explain this phenomenon: requiring active protein synthesis and metabolism for ROS generation, ROS accumulation with bacterial colony growth after quinolone removal, and occurrence within senescent cells exposed to quinolones [141]. Non-quinolone ROS inhibitors (e.g., thiourea) were also shown to inhibit bacterial death, while defects in ROS detoxification enzymes (e.g., catalase) enhanced bacterial death. These findings were refined to suggest a dual role of ROS in bacterial death and adaptation, with future implications for antibiotic resistance and tolerance mechanisms [142]. Li et al. highlighted the framework for a self-amplifying intracellular ROS cascade triggered by bactericidal antibiotics in combination with host immune responses. This secondary function of quinolones and other established bactericidal antibiotics relies on the premise of ROS-mediated intracellular metabolic dysregulation, ETC overactivation, and amplification of oxidative stress once a critical threshold concentration of ROS is reached. However, upregulation of antioxidant enzymes (e.g., catalase and SOD) along with shunting energy-storing substrates towards alternate metabolic pathways (e.g., pentose phosphate pathway and glyoxylate shunt) was demonstrated to mitigate bacterial intracellular oxidative stress. Continuous sublethal ROS concentration exposure, therefore, may promote bacterial mutation and thus bacterial resistance to antibiotic treatments [142].

The paradox of bacterial survival in these conditions presents another fascinating direction for future study of antioxidant therapeutic targets. Although the direct role of ROS in bactericidal lethality has yet to be fully unraveled, current studies are challenging prior assumptions held for imbalances in oxidative stress and cell survival [143]. Considering the well-established antioxidant and signaling properties of VD and the VDR, we suspect the addition of VD to future in vitro models could attenuate the effectiveness of certain antibiotics in toxic conditions of oxidative stress. Quinolone-based antibiotics, which are used to treat a large family of infectious diseases, have also been experimentally observed to display heterogeneity in resistance patterns and bactericidal efficacy when administered in conjunction with ROS-producing metabolites in* E. coli* and *Mycobacterium tuberculosis *[144,145]. As other classes of antibiotic agents display similarly complex heterogeneous interactions with ROS, the variability in resistance of this class of antibiotics opens the possibility of modulation by supplemental antioxidant therapies to reduce resistance or increase susceptibility of the targeted bacteria to quinolones. These unknown mechanisms present additional opportunities to study the present utility of these antibiotics and the possible benefit of synergism from antioxidant agents such as VD.

## Conclusions

VDD and VD supplementation have profound and intricate relationships with the pathophysiology underlying diseases and disorders of oxidative stress. Mitochondrial function and its roles in reduction-oxidation balance, apoptosis, detoxification, and respiration are implicated in every organ system discussed within this review and remain a focus for directed antioxidant interventions. Crucially, perturbations in the delicate and complex system of oxidative homeostasis and resultant ROS production are the key driving force behind the myriads of pathological conditions exacerbated by VDD and thus the potential for applicability of VD treatment. Management of ROS using VD and adjacent antioxidant therapies is well documented in scientific and medical literature, as evidenced by the reviews presented. Although experimental support of VD supplementation in specific disease courses, such as neurodegenerative diseases and sleep disorders, remains inconclusive in delineating the most effective utilization of VD, evidence is growing toward understanding the appropriate role of antioxidant reversal to limit disease progression. Furthermore, the role of VDD in the development of many of these conditions discussed is foundational in understanding the broader scope of mineral deficiencies, their regulation of the delicate balance in reduction-oxidation physiology, and the progression of cellular aging. Summarization of these aspects of the current scientific literature and presenting the potential roles of VD in slowing the progression of oxidative disease warrants ongoing investigation as the chronicity of these conditions becomes more commonplace.
